# Caging giants: Characterizing the molecular mechanisms of neutrophil swarming against *Candida albican*s hyphae

**DOI:** 10.1093/jleuko/qiaf082

**Published:** 2025-06-06

**Authors:** Tasha K Phillips, Kelsey Lawson, Tammy R Ozment, Allison Scherer, Alex Hopke

**Affiliations:** Department of Biomedical Sciences, East Tennessee State University Quillen College of Medicine, PO Box 70577, Johnson City, TN 37614, United States; Department of Natural Sciences, University of Virginia’s College at Wise, 1 College Ave, Wise, VA 24293, United States; Department of Surgery, East Tennessee State University Quillen College of Medicine, PO Box 70575, Johnson City, TN 37614, United States; Center of Excellence in Inflammation, Infectious Disease and Immunity, East Tennessee State University, PO Box 70422, Johnson City, TN 37614, United States; Department of Natural Sciences, University of Virginia’s College at Wise, 1 College Ave, Wise, VA 24293, United States; Department of Biomedical Sciences, East Tennessee State University Quillen College of Medicine, PO Box 70577, Johnson City, TN 37614, United States; Center of Excellence in Inflammation, Infectious Disease and Immunity, East Tennessee State University, PO Box 70422, Johnson City, TN 37614, United States

**Keywords:** fungal infection, hyphae, neutrophil, swarming

## Abstract

Neutrophils utilize many mechanisms to restrict fungal growth. When phagocytosis occurs, neutrophils can create many toxic antimicrobials including reactive oxygen species and the products of myeloperoxidase. If a pathogen is too large to phagocytose, neutrophils can also resort to the release of neutrophil extracellular traps or it can engage in the behavior of “swarming,” in which the recruitment and antimicrobial action of many neutrophils are coordinated against a single target. Here we optimized an assay to study the behavior of swarming directly against live *Candida albicans* hyphae. We find that hyphae are highly potent targets for inducing swarming behavior and that swarming is very effective at restricting hyphal growth. We provide insight into the initial interactions between the pioneer neutrophil and the hyphae, including information on how fast signaling is initiated following neutrophil binding, how far neutrophils stretch before signaling occurs, and how the calcium signaling waves are unique in response to hyphal targets. We also find distinct and important roles for myeloperoxidase, spleen tyrosine kinase, Bruton's tyrosine Kinase, and CD18 in an effective neutrophil swarming response.

## Teaser

Microscale arrays enabled the characterization of the neutrophil swarming response to the hyphae of *Candida albicans* as well as provided insights into the molecular mechanisms that drive and regulate the process.

## 1. Introduction

Neutrophils are a critical effector cell of the innate immune system and are especially important for host defense against infection. Neutrophils have a wide arsenal of antimicrobial mechanisms with which they can use to try to kill microbes, including the generation of reactive oxygen species (ROS) or reactive nitrogen species, the action of myeloperoxidase (MPO), antimicrobial peptides, and degradative enzymes like neutrophil elastase and cathepsin G.^1,2^ This arsenal has long been characterized and is usually thought of in the context of being deployed following recognition and phagocytosis of the invading microbes. Recently, it has been recognized that neutrophils can sense the size of the target and can deploy other, additional, options if the target is too large to be phagocytosed. These include deploying their own DNA, in the form of sticky neutrophil extracellular traps (NETs), as well as using the more recently characterized behavior of neutrophil swarming.^3–8^ During swarming, the actions of many neutrophils are coordinated against a single target to allow them to concentrate their microbicidal activity against targets that cannot be addressed by individual cells and to insulate healthy tissue.^7,8^

Recent work has begun to characterize the molecular mechanisms that drive swarming biology in detail. While in vivo models in the mouse and the zebrafish have provided critical insights, they are low throughput and may not totally translate to human cells.^5,9–11^ To complement these models and drive the field forward more rapidly, we and others have developed and leveraged in vitro models like microscale arrays of microbial clusters.^10,12–14^ These systems allow for the direct observation of swarming behavior against many targets simultaneously and for many conditions to be tested in parallel, dramatically increasing throughput. Importantly, this system can be used with cell lines and mouse or human neutrophils, allowing for the comparison of cross-species differences as well as the ability to directly explore swarming behavior in human cells.^10,12,13,15,16^

Swarming may be particularly important during infection, as this behavior allows neutrophils to coordinate and concentrate their microbicidal activities against a single target and to seal in pathogens, thereby preventing their invasion of nearby healthy tissue. Swarming has been observed in response to many pathogens including bacteria, protozoans, helminths, and fungi.^5,11,13,16–19^ However, the molecular determinants of these interactions and their consequences for the outcome of infection remained poorly characterized and may be context dependent. For example, recent work in mouse models has suggested that neutrophil swarming plays an important role in defense against *Candida albicans* tissue invasion during oral infection; however, swarming to *C. albicans* in the vasculature of the lung has led to negative outcomes for the host.^18,20^ To further characterize the molecular mechanisms involved in swarming against *C. albicans*, microscale arrays using live clusters of *C. albicans* yeast were leveraged to study the mechanisms deployed by human neutrophils upon encountering these pathogens.^13^ We found that swarming required LTB_4_ and ROS signaling to effectively regulate swarm dynamics and that ROS, MPO, and NET production all contributed to restricting fungal growth.^13^ These results demonstrated that swarming had potent antifungal potential and provided a starting point for molecular insights into the mechanisms required for this process. Unfortunately, much remains poorly characterized about the process of swarming and its role during infection and inflammation.

Furthermore, there has been some question about the relevance of these results to what will actually be encountered during infection in vivo as, outside of biofilms, it may not be common for the immune system to encounter large tightly packed clusters of yeast. However, many pathogenic fungi, including *C. albicans*, are capable of morphological switching from yeast to grow as much larger hyphae. These hyphae can be many times larger than a neutrophil and therefore unable to be phagocytosed, so they would represent a potential swarming target (i.e. highly relevant and present during infection in vivo).^21^ Furthermore, yeast and hyphae are not identical for reasons beyond just the sizes of each morphology, with changes in the cell wall component contents and organization as well as differential expression of many genes, proteins, and secreted factors.^22–25^ We therefore cannot simply assume that the swarming responses to yeast clusters will directly translate to hyphae. While neutrophils are known to be important for killing hyphae, the role of swarming in this process remains largely uncharacterized.^26,27^ Hyphae are critical for virulence for many fungal infections, including those by *C. albicans*, so elucidating the role of swarming in the immune response to this morphology may highlight another important host defense pathway that can be leveraged for future therapeutic development.^21,24^

Here, we build on previous results looking at neutrophil swarming and extend these tools to look directly at the hyphae of *C. albicans*. We demonstrate that hyphae are a potent inducer of swarming responses in human neutrophils. By leveraging the Calbryte fluorescent probe (AAT Bioquest), which was recently shown to allow visualization of calcium involved swarming initiation signaling waves, we examined the early events of swarming against hyphae in unprecedented detail.^28^ We characterized several mediators required for effective swarming to hyphae including MPO function, as well as spleen tyrosine kinase (SYK) and Bruton's tyrosine kinase (BTK) signaling. We also uncover a novel role for CD18 in effective swarm formation and function against hyphae. Taken together, these results demonstrate that these microscale arrays continue to be an effective tool to probe the molecular mechanisms that drive swarming biology and provide important insights into the swarming response directly against *C. albicans* hyphae, a biologically relevant pathogenic target.

## 2. Methods

### 2.1 Array printing

Utilizing a microarray printing platform (Picospotter; PolyPico), we printed a solution of 0.1% poly-L-lysine (Sigma-Aldrich) and ZETAG, sometimes with a small amount of poly-L-lysine FITC included to enable fluorescent screening of arrays. For experiments, 8 × 8 arrays in 16-well formation were printed on glass slides (Fischer Scientific), with individual spots around 100 µm diameter.^12^ Following printing, slides were screened for accuracy and dried at room temperature or at 37 °C for approximately 3 h.

### 2.2 Neutrophil isolation

Protocols were reviewed and approved by the Institutional Biosafety and Chemical Safety Committee at East Tennessee State University. Fresh samples of peripheral whole blood from healthy volunteers were purchased from a local location of BioIVT. Samples were collected in EDTA tubes in accordance with the protocols approved by the Institutional Review Board at BioIVT and were de-identified before being provided for use. Human neutrophils were isolated from these samples using the EasySep Direct Human Neutrophil Isolation Kit per the manufacturer's protocol (STEMCELL Technologies) within 6 h of blood draw. For applicable experiments, isolated cells were stained with Hoechst, washed once, and then resuspended in Iscove's Modified Dulbecco's Media (IMDM) with 20% fetal bovine serum (FBS) (Thermo Fisher Scientific).

### 2.3 Microorganism strains and culture

*C. albicans* constitutively expressing a far-red fluorescent protein was inoculated into fresh yeast extract peptone dextrose liquid media and grown on a rotational incubator at 30 °C overnight.^29^ Yeast were transferred to 5 mL of RPMI 1640 (Thermo Fisher Scientific) and incubated on a rotational incubator at 30 °C for approximately 16 h. Cultures were then placed on ice until use.

### 2.4 Target patterning

After drying, 16-well ProPlate wells (Grace Bio-Labs) were attached to glass slides with printed arrays. Hyphal cultures were diluted 1:1 in sterile water, and 50 µL of the desired target was added to each well and incubated with rocking for 3 min. Following incubation, wells were washed out with sterile water to remove unbound targets from the surface of the glass. Each slide was then screened to ensure proper patterning of targets onto the printed grids. Following addition of microbial targets, wells were coated in fibronectin (R&D Systems) at 10 µg/mL for 30 min at 37 °C. Wells were then washed out and had 50 µL of phosphate-buffered saline added to prevent drying out until used.

### 2.5 Swarming experiments

Time-lapse imaging studies were executed using the Leica TCS SP8 confocal microscope (software: LAS-X) with a motorized stage. Wells were maintained at 37 °C throughout the course of imaging using an environmental chamber (Okolab). Swarming targets were chosen at 10× magnification (Numerical aperture [NA] = 0.4) and marked using the multipoint function prior to loading the neutrophils into each well (5 × 10^5^). All selected points were optimized using the software's autofocus function before launching each experiment, and autofocus was used throughout imaging. In experiments using antibodies or chemical inhibitors, neutrophils were incubated for 1 h at 37 °C with appropriate antibody or inhibitor prior to use. During experiments with Calbryte, hyphal swarming targets were selected at 10× magnification (NA = 0.4) and an additional digital zoom was applied at 1.7×. Endpoint images were taken at 4x magnification (NA = 0.16) or 20× magnification (NA = 0.8) using the Olympus BX63 microscope (software: Cellsens) the following day. Bright-field and fluorescent images were obtained using the Olympus DP74 and Hamamatsu ORCA flash 4.0LT (C11440), respectively.

### 2.6 Fluorescent probes and chemical or antibody inhibition

For longer experiments, neutrophils were stained with Hoechst, and to visualize NET formation, SYTOX Green (Thermo Fisher Scientific) was included at 0.25 µM. For short, high-temporal-resolution experiments, neutrophils were instead stained just with Calbryte at 1 µM. For inhibition of MPO activity, we included 4-ABAH in the media at a concentration of 50 µM (Cayman Chemicals). For inhibition of SYK, we included PRT060318 in the media at a concentration of 4 µM (Cayman Chemicals). For inhibition of BTK, we included acalabrutinib at 1 µM (Cayman Chemicals). To act as LTB_4_ receptor antagonists, we used BIIL-260 hydrochloride (MedChemExpress), CP-105696 (MedChemExpress), or SB-209247 (MedChemExpress) at 10 µM. Neutrophils were incubated for 30 min at 37 °C. For NETosis inhibition, we used GSK 484, a PAD4 inhibitor (Abcam) at 10 µM. Neutrophils were incubated at 37 °C for 30 min. Cells were then spun down and GSK 484 was removed. Cells were resuspended in IMDM + 20% FBS as previously described. For inhibition of CD18 (clone: TS1/18), CD11a (clone: HI111), CD11b (clones: VIM12, ICRF44, or M1/70) or CD11c (clone: 3.9) (BioLegend or Invitrogen for VIM12), we incubated neutrophils with the appropriate antibody at 20 µg/mL for 60 min at 37 °C. The isotype controls IgG1 (clone: MOPC-21) and IgG2b (clone: RTK4530) (BioLegend) were used at the same concentration. All antibodies used in this study underwent dialysis overnight using Slide-A-Lyzer Dialysis cassettes (Thermo Fisher Scientific), 10,000 molecular weight cutoff, prior to use to remove sodium azide.

### 2.7 Flow Cytometry

Phagocytosis and ROS production were measured by flow cytometry on a BD LSRFortessa X-20 running BD FACSDiva software (BD Biosciences). Briefly, human neutrophils were isolated from blood, co-incubated for 20 min at room temperature with media only, isotype control, or CD18 antibody. Neutrophils were brought to 5 × 10^5^ cells/mL in 500 µL in IMDM + 20% FBS and co-incubated with *C. albicans* (SC5314-iRFP) yeast at a multiplicity of infection of 5 or 2, dihydrorhodamine 123 (Thermo Fisher Scientific; 5 µM final concentration), and cytochalasin D (Thermo Fisher Scientific; 30 µM final concentration) where indicated. Neutrophils and yeast were co-incubated for 20 min at 37 °C on 5% CO_2_ with the tubes placed on their sides. Samples were then placed on ice, washed with FACS buffer (2% heat-inactivated FBS in phosphate-buffered saline), labeled with PE anti-mouse/human CD11b antibody (BioLegend; clone M1/70) during CD18 inhibition experiments or PE anti-mouse/human CD66b antibody (BioLegend; clone 6/40c) for CD11b inhibition experiments, and washed before flow cytometry. Neutrophils were gated by forward scatter and side scatter and then for CD11b-postive or CD66b-positive cells. Neutrophils that had engulfed *C. albicans* were found by percent iRFP fluorescence in the AF700 channel, and neutrophils producing ROS were found by percent dihydrorhodamine 123 fluorescence in the FITC channel.

### 2.8 Killing Assay

The killing assay was performed as described previously, with some minor adjustments.^30^ Briefly, human neutrophils were harvested 1 d post draw and co-incubated for 30 min at room temperature with media only, isotype control, or CD18 antibody. Neutrophils were then plated as 5 × 10^4^ cells/mL neutrophils per well in IMDM + 20% FBS and challenged with *C. albicans* yeast at a multiplicity of infection of 5 or 2 for 2 h at 37 °C. The plate was moved to ice immediately after challenge, and neutrophils were lysed with Nonidet P40 (final concentration 1%). MOPS-RPMI and PrestoBlue (Thermo Fisher Scientific) were added to each well, and the plate was incubated at 37 °C for 2 h when the fluorescent endpoint read was taken on a BioTek Cytation 1 plate reader (Agilent; software version 3.12.08). The fluorescence from each technical triplicate was compared with the *C. albicans* only control wells with percent killing determined with the following formula: 1 − (viability of yeast + neutrophil condition/viability of yeast only condition).

### 2.9 Swarming analysis

Time-lapse images were manually analyzed using FIJI (Fiji is just ImageJ v2.9.0/1.53t; National Institutes of Health).^31^ The area analysis was performed by manually outlining the swarms or areas of fungal growth.^12,13^ For area of the swarm, only the neutrophils themselves were measured, using the DAPI fluorescent channel images, with Hoechst staining to identify the neutrophils for analysis. For areas of fungal growth, a combination of bright-field and fluorescent channels were used. We combined the appropriate fluorescent channel with the bright-field image to be sure we included any escaped fungal elements, like lone hyphae, that may not show up well in the fluorescent channel. Intensity profiles of SYTOX Green staining were generated by defining regions of interest in the FITC channel and using the time measurement option in the FIJI software. For analysis of Calbryte images, images in the FITC channel were used. Calbryte signaling was examined after polymorphonuclear neutrophil (PMN) attachment to hyphae, and signaling was defined as increase in fluorescence from baseline after this time.

## 3. Results

Building on our previous arrays for yeast clusters, we adapted the system for pregrown hyphae. The hyphae of *C. albicans* were grown for the indicated amount of time prior to incubation with the poly-L-lysine printed arrays and allowed to adhere, creating large arrays of spots with small numbers of hyphae on each (Fig. S1). As with the yeast clusters shown previously,^13^ these hyphae were viable and grew well on the arrays (Fig. S1). By adjusting the concentration, we can change the number of hyphae on the spots in the array, ranging from just a few separated hyphae to large clusters of hyphae (not shown).

**Fig. 1.**
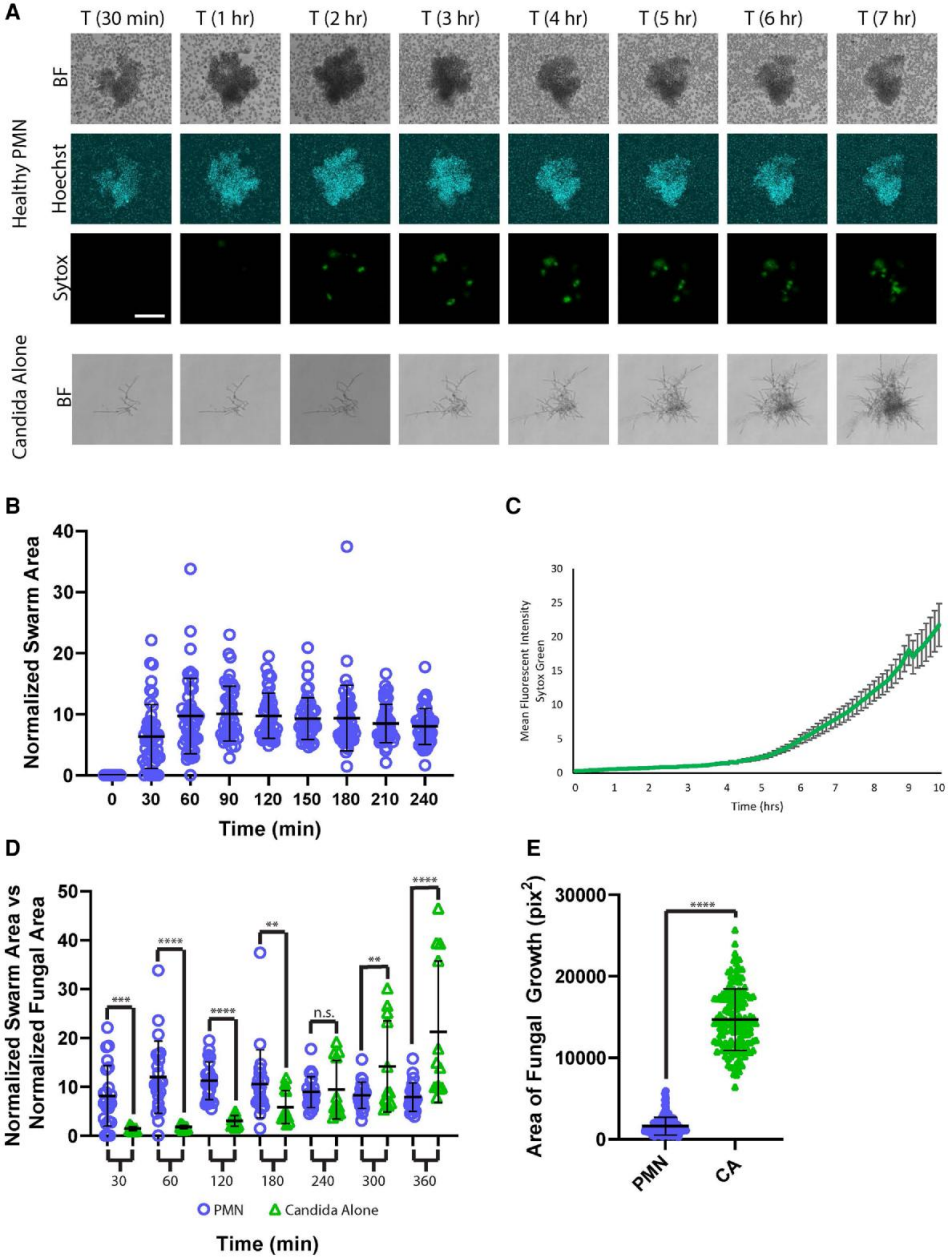
*Candida albicans* hyphae are potent inducers of neutrophil swarming and swarming effectively restricts hyphal growth. Live *C. albicans* hyphae were patterned on poly-L-lysine/ZETAG arrays and incubated with purified human neutrophils or alone in media. (A) Time-lapse images show the progression of neutrophil swarms or *C. albicans* hyphal growth over time. Bright-field (BF), DAPI (Hoechst), and FITC (SYTOX Green) channels are represented. (B) The area of each neutrophil swarm was quantified at the indicated times and normalized the area of the hyphae at that spot. There were 47 swarms across 6 donors. (C) The mean fluorescence intensity of SYTOX Green at each swarm over time was quantified. There were ≥38 swarms across 6 donors. (D) The area of spot of hyphae growing alone in media, normalized to the starting amount of hyphae, was quantified and compared with the normalized growth of neutrophil swarms from panel B. There were 23 for swarms and 12 for the *Candida* alone from 3 donors. (E) The remaining fungal growth was quantified after 16 h of incubation with human neutrophils or *Candida* and media only (CA). There were ≥146 spots across 6 donors. **P ≤ 0.01, ***P ≤ 0.001, and ****P < 0.0001 by Student’s *t* test or Mann-Whitney test. Error bars represent standard deviation except in panel C, in which they represent SEM. Scale bar = 100 µm. n.s., not significant; PMN, polymorphonuclear neutrophil.

We next leveraged this system to monitor the interactions of human neutrophils directly with these fully formed fungal hyphae. We found that, despite representing less overall fungal mass than yeast clusters, hyphae were able to elicit robust swarming responses (Fig. 1), confirming that hyphae are biologically relevant and highly inflammatory swarming targets. As the number of hyphae and hyphal size from spot to spot was more heterogeneous than the generally identically sized yeast clusters, we normalized the area of each swarm to the total starting fungal area of the hyphae on that individual spot. Swarming dynamics were similar to those observed against yeast clusters, with exponential recruitment of neutrophils to the target within the first 30 min, followed by a plateau in cluster size by 2 h (Fig. 1B; Movie S1). Treatment with LTB_4_ receptor antagonists resulted in significant, though not complete, reductions in swarm size and fungal control (Fig. S2). We also included SYTOX Green in the media to examine cell death within the swarms against hyphae, something that we found previously to be indicative of NETs against the yeast clusters.^13^ Interestingly, we found a distinct SYTOX Green profile in the swarms against hyphae showing that, while the SYTOX signal increases over time as seen for yeast clusters previously, the signal seen with hyphae was slower to appear and remained lower for many hours.^13^ When compared head to head, it was clear that yeast clusters elicited substantially more SYTOX Green fluorescence inside their swarms than the swarms against hyphae (Fig. S3A) until 8 h or later (Fig. 1C; Fig. S3A). While SYTOX Green itself is not specific for NETs, we found that the SYTOX Green signal was minimal when neutrophils were not present and that treatment with the PAD4 inhibitor, GSK484, reduced this signal, suggesting they are visualizing NETs (Fig. S3C–F). We found that swarming was highly effective at restricting the growth of hyphae, even more so than for the yeast clusters (Fig. 1D, E; Fig. S3B). On average, hyphae left alone in media grew significantly beyond the average size of a neutrophil swarm within 5 h (Fig. 1D). This restriction lasted long term, with swarming keeping fungi restricted to an average size approximately 9 times smaller than if the hyphae were allowed to grow alone for 16 h (Fig. 1E). This restriction showed a small, but significant, defect during PAD4 inhibition (Fig. S3D).

**Fig. 2.**
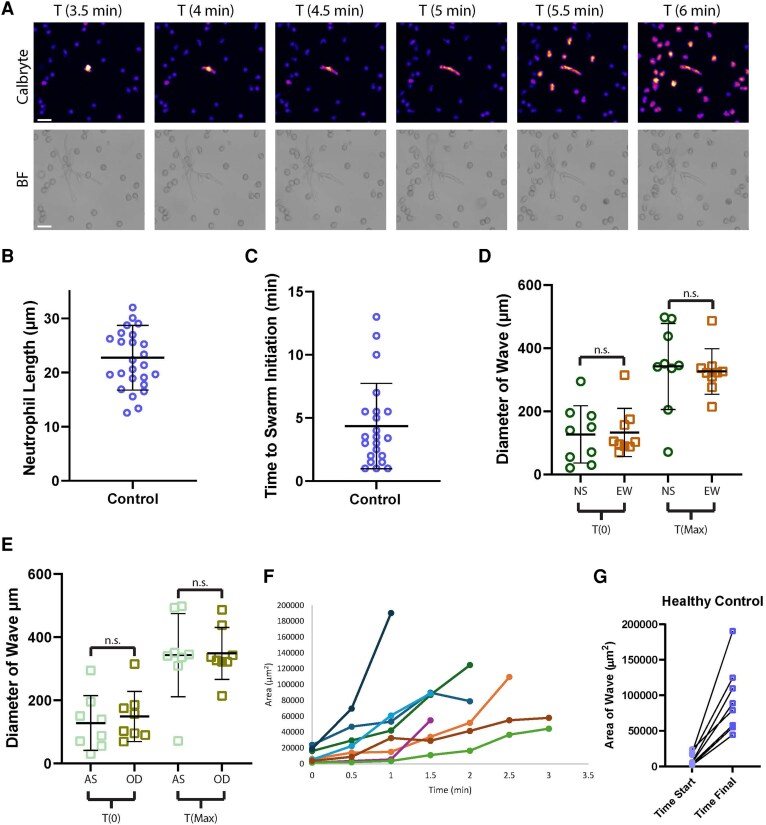
Calbryte staining reveals insights into pioneer neutrophil initiation events. Neutrophils were stained with Calbryte prior to addition to arrays of live *Candida albicans* hyphae. (A) Representative time-lapse images show the interaction between a Calbryte-stained pioneer neutrophil and hyphae. Bright-field (BF) and FITC (Calbryte) channels are shown. (B) The length the neutrophil stretched before the Calbryte signal spreads to neighboring cells was quantified. There were 26 neutrophils across 9 donors. (C) The time from the attachment of the neutrophil to the hyphae until the Calbryte signal spread to neighboring cells was quantified. There were 22 neutrophils across 8 donors. (D) The diameter of the Calbryte positive signaling wave was measured at the first frame of wave spread and at the frame where the wave spread to its maximum size. The diameter was measured for both the north/south axis (NS) and the east/west axis (EW). There were 9 waves across 4 donors. (E) The diameter of the Calbryte positive signaling wave was measured at the first frame of wave spread and at the frame where the wave spread to its maximum size. The diameters were then sorted by if they were the along the axis the neutrophil attached from (AS) or if they were the opposite axis (OD). There were 8 waves across 4 donors. (F) The area covered by the wave was calculated from the first frame it spread from the pioneer neutrophil until its maximum size. Each line represents an individual wave. (G) The area covered by each wave in the first visible frame (time start) and at its maximum size (time final) are shown. There were 8 waves across 4 donors. One-way analysis of variance with Tukey's posttest for panel E or Kruskal-Wallis test for panel D. Error bars indicate standard deviation. n.s., not significant. Scale bar = 50 µm.

Recently, it was shown that the use of the fluorescent probe Calbryte, which visualizes the influx of calcium into the cytosol of the cell via increased fluorescence, could allow the visualization and quantification of the activation of neutrophils and the initiation signaling waves in the swarming response against yeast clusters.^28^ We therefore added this probe to our assay to gain insights into the early events of swarming against hyphae (Fig. 2; Movie S2). As the hyphae presented a single target for neutrophil interaction vs a cluster of 100+ individual yeasts, we were able to gain important insights into these early initiating events. With hyphae being a single target, it was easier to identify which neutrophils were the pioneer cells that encountered and attached to the fungus first. By monitoring these pioneer neutrophils and incorporating the Calbryte probe, we could directly quantify early events in the swarming response, such as the time from attachment to the fungus to the initialization of swarming, which we defined as the activation of Calbryte signal that spread out to neighboring cells as a wave, like those defined previously.^28^ We found that the first responding neutrophils took an average of 4.39 min from time of attachment to the hyphae until the initialized the swarming response (Fig. 2A–C). There was heterogeneity in this response, but with activation occurring in as little as 1 min and taking up to 13 min, suggesting that this early event is flexible and may be influenced by other outside factors (Fig. 2C).

Swarming has been hypothesized to be particularly relevant as a response to large pathogens and previous work using zymosan or yeast clusters has shown that target size influences the induction of swarming response, with targets below a threshold not inducing swarming.^10,13^ By monitoring these first responding neutrophils, we could also directly measure how far they stretched during attachment to fungal hyphae before they sent out the signal to initialize swarming, again defined as Calbryte signaling that spread to neighboring cells, as seen previously.^28^ We found that neutrophils stretched an average 22.74 µm prior to sending out the signal (Fig. 2B). Interestingly, there was also heterogeneity found here, with the minimum stretching length being 12.55 µm and the maximum seen being 36.92 µm stretched before swarming initiation.

We also leveraged the Calbryte probe to measure the waves of neutrophil activation during swarming initiation. We found that the initial Calbryte signaling wave spread to an average diameter of 334.15 µm before terminating or being overcome by secondary waves (Fig. 2D, E). These waves were relatively symmetric, as we measured the diameter of the waves in both the north/south axis and the east/west axis at both initiation and termination and did not find significant differences in either direction (Fig. 2D). The wave size did not seem to be influenced by the direction the neutrophil was facing during attachment, as there were not significant differences in the diameter of the wave when the side the neutrophil attached to was compared with the opposite axis (Fig. 2E). We also calculated the area covered by the initial signaling wave over time to examine the dynamics of individual waves. Calbryte signaling waves covered an average area of 93,794 um^2^ over a period of 3 min or less before terminating, though there was significant heterogeneity (Fig. 2F, G).

**Fig. 3.**
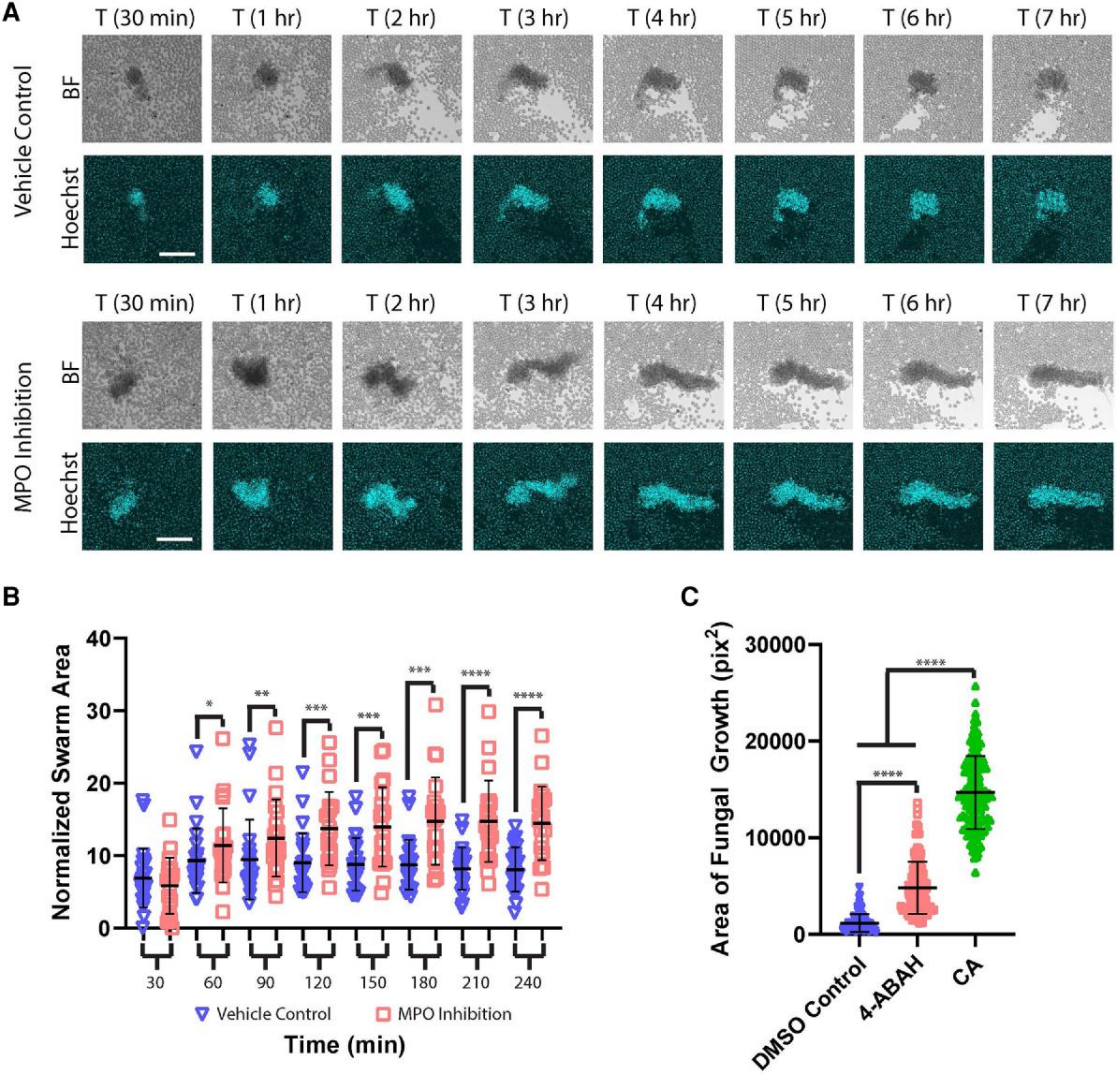
MPO regulates swarm dynamics and antimicrobial effectiveness. Human neutrophils were incubated with the MPO inhibitor 4-ABAH or vehicle control and then added to the *Candida albicans* hyphae arrays. (A) Time-lapse images show the progression of swarming in controls and during MPO inhibition. Bright-field (BF) and DAPI (Hoechst) channels are shown. (B) The area of the swarms at the indicated time was quantified and normalized to the amount of starting hyphae. There were ≥22 swarms across 6 donors. (C) The remaining fungal growth was quantified after 16 h of incubation with human neutrophils treated with 4-ABAH, vehicle control, or *Candida* and media only (CA). There were ≥101 spots across 3 donors. *P ≤ 0.05, **P ≤ 0.01, ***P ≤ 0.001, and ****P < 0.0001 by Mann-Whitney or Kruskal-Wallis test. Error bars represent standard deviation. Scale bar = 100 µm. DMSO, dimethyl sulfoxide.

We next leveraged these arrays to probe and characterize the molecular mechanisms that drive swarming responses and allow them to restrict fungal hyphae. In agreement with previous results for yeast clusters, we found a critical role for MPO function in the effective restriction of hyphal growth, as inhibition with the chemical inhibitor 4-ABAH resulted in significant defects (Fig. 3A–C). We also found that MPO inhibition resulted in much larger swarm sizes than controls (Fig. 3B). Previously, we and others have shown that ROS plays a role regulating swarm dynamics, as ROS inhibition resulted in larger, but less effective, swarm formation.^13,28^ However, these results were usually obtained by directly targeting NADPH oxidase function, so our results here extend these findings to suggest that the products of MPO function can also play a role in regulating swarm dynamics. Despite this, these larger swarms remained compromised in their ability to restrict *C. albicans* hyphae (Fig. 3C). There did not appear to be major differences in the SYTOX Green signal between control and 4-ABAH conditions (Fig. S4).

**Fig. 4.**
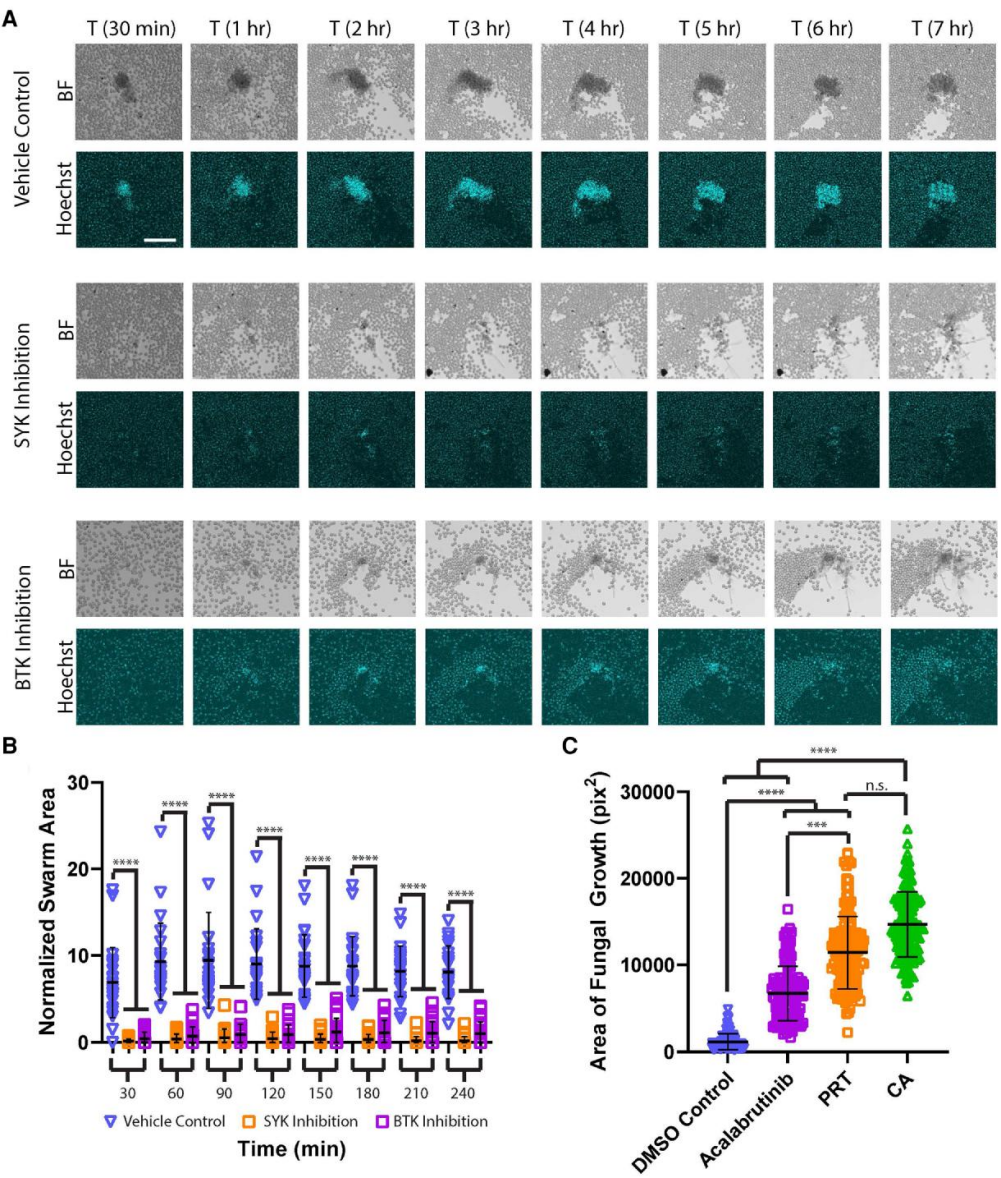
SYK and BTK inhibition compromise swarming and fungal restriction. Human neutrophils were incubated with the SYK inhibitor PRT, the BTK inhibitor acalabrutinib, or vehicle control and then added to the *Candida albicans* hyphae arrays. The vehicle control is the same as in Figure 3, as these conditions were all run in the same experiments. (A) Time-lapse images show the progression of swarming in controls and during SYK or BTK inhibition. Bright-field (BF) and DAPI (Hoechst) channels are shown. (B) The area of the swarms at the indicated time was quantified and normalized to the amount of starting hyphae. There were ≥23 swarms across 6 donors. (C) The remaining fungal growth was quantified after 16 h of incubation with human neutrophils treated with SYK or BTK inhibitor, vehicle control, or media only. There were ≥101 spots across 3 donors. ***P ≤ 0.001 and ****P < 0.0001 by Kruskal-Wallis test. Error bars represent standard deviation. Scale bar = 100 µm. n.s., not significant.

**Fig. 5.**
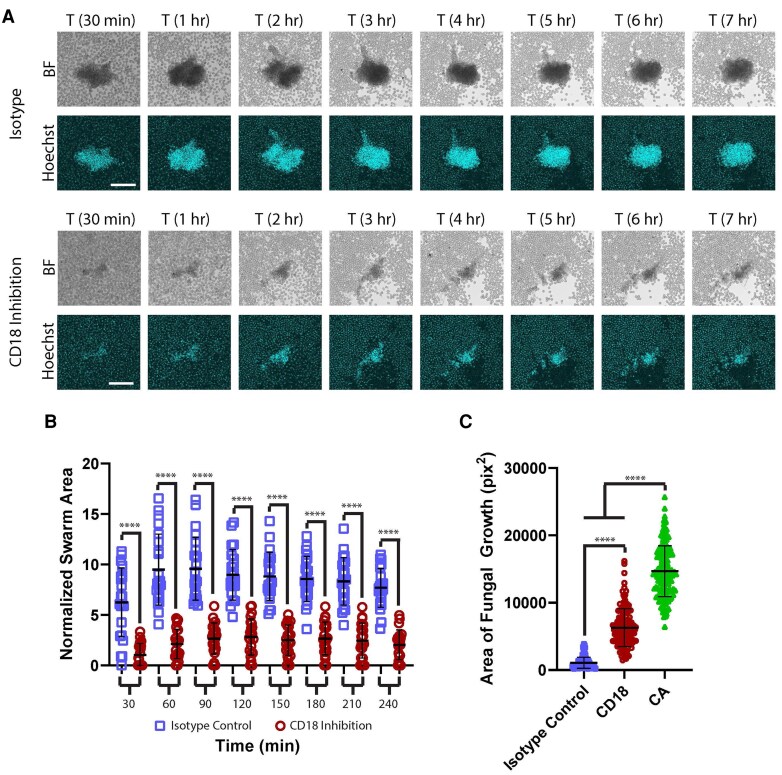
CD18 is critical for effective swarm formation and hyphal restriction. Human neutrophils were incubated with the CD18 blocking antibody or isotype control and then added to the *Candida albicans* hyphae arrays. (A) Time-lapse images show the progression of swarming in controls and during CD18 inhibition. Bright-field (BF) and DAPI (Hoechst) channels are shown. (B) The area of the swarms at the indicated time was quantified and normalized to the amount of starting hyphae. There were 22 swarms across 6 donors. (C) The remaining fungal growth was quantified after 16 h of incubation with human neutrophils treated with CD18 blocking antibody, isotype control, or *Candida* and media only (CA). There were ≥121 spots across 3 donors. ****P < 0.0001 by Kruskal-Wallis test. Error bars represent standard deviation. Scale bar = 100 µm.

We also investigated which signaling cascades controlled these swarming responses. Previously, it was seen that SYK was a critical regulator of responses to yeast clusters and that BTK was important for responses to clusters of *Aspergillus fumigatus* conidia.^30,32^ We therefore explored if the function of these mediators would be conserved in regulating the response to *C. albicans* hyphae. We found that SYK signaling did indeed play a critical role in swarming responses to hyphae (Fig. 4). During SYK inhibition with the chemical inhibitor PRT, we found that swarming responses were almost completely abolished compared with controls (Fig. 4B). This resulted in a profound defect in the restriction of hyphal growth, allowing fungal growth to proceed to the point where it was not significantly different from the *Candida* alone controls, which were not subject to any neutrophil attack (Fig. 4C). BTK inhibition by the chemical inhibitor acalabrutinib revealed that BTK also plays an important role in swarming response to these hyphae. BTK inhibited neutrophils also showed significant defects in swarm formation compared with controls, although there was a trend toward larger cluster formation than SYK-inhibited controls (Fig. 4B). BTK inhibition also resulted in significant defects in the swarm's ability to restrict fungal growth, but these defects were not as significant as those seen during SYK inhibition (Fig. 4C). We also examined early signaling wave and initiation events using Calbryte staining during SYK and BTK inhibition. We found that treatment with these inhibitors either completely prevented the initiation and spread of a wave (particularly for SYK inhibition) or resulted in waves that traveled much shorter distances than those seen in healthy controls (Fig. S5A–E). This led to the recruitment of neutrophils over greatly reduced distances compared with healthy controls, which resulted in significantly smaller swarms (Fig. 5; Fig. S5B). Together, these results confirm important roles for both SYK and BTK in regulating swarming responses and extend this specifically to the response against *C. albicans* hyphae.

We also began work to examine which cell surface receptors play critical roles in regulating the swarming response. During this work we have identified a critical role for CD18 in effective swarm function. Neutrophils treated with blocking antibodies directed against CD18 showed significant defects in the growth of swarm clusters over time (Fig. 5). Unlike the SYK-inhibited neutrophils, some clustering does occur; however, it appears that once the first wave of neutrophils arrive and cover the hyphae, further neutrophils are unable to join the cluster (Fig. 5A). This contrasts with what occurs in controls, in which further neutrophils pile on into the growing swarm, growing to an average 6 times larger than CD18 inhibited clusters at even 30 min and remaining an average of 3.7 times larger by 4 h from the start of the assay (Fig. 5B). Interestingly, CD18 inhibition did not cause significant changes to early initiation events in swarming, as measures like time from attachment to initiation, neutrophil stretching, and wave size were not significantly different when compared with isotype controls, which formed normal effective swarms (Fig. S6A–E). CD18 inhibition also results in serious defects in the ability of the swarm to restrict fungal growth of the hyphae (Fig. 5C). These specific defects appear to be unique to CD18, as inhibition of known interacting partners of CD18 including CD11a, CD11b, and CD11c all did not show this phenotype (Figs. S7 and S8). For the inhibition of CD11a or CD11c, no significant differences from isotype control for swarm size or fungal control were found (Fig. S7). For inhibition of CD11b, we tested multiple clones and received different phenotypes (Fig. S8). For clone M1/70, we did not see any significant changes, and for clone ICRF44 we saw no significant changes except at the final timepoint of swarming, where inhibited swarms were bigger than controls. For both of these clones, no changes to fungal control were seen (Fig. S8A–D). For the final clone, VIM12, we saw significant changes to swarming. Treatment with this clone caused neutrophils to cluster throughout the field of view, but these smaller clusters still migrated in a directed fashion into the larger swarm against the hyphal targets (Fig. S8E; Movie SX). This resulted in swarms that were significantly larger throughout the assay. Interestingly, fungal growth was also increased during inhibition by this clone, despite the swarms being larger (Fig. S8F). Despite this, inhibition with any of the CD11b clones did not result in a phenotype that resembled that seen during CD18 inhibition (Fig. 5; Fig. S8). We also tested these clones in a multiparameter flow assay that examined phagocytosis and ROS production against yeast, in which swarming would not occur. We found that they did not cause significant defects in phagocytosis or ROS production compared with the isotype or untreated controls in these conditions (Fig. S9). Finally, we tested if the role of CD18 in fungal killing was unique to swarming by employing the same multiparameter flow assay that examined phagocytosis and ROS production against individual yeast, as well as a traditional plate-based killing assay. We found that inhibition of CD18 in this assay did not have significant impacts on phagocytosis, ROS production, or killing of yeast, demonstrating that our observed phenotype is unique to swarming in these conditions (Fig. S10A–C).

## 4. Discussion

We have optimized our microscale swarming arrays to facilitate the study of human neutrophil interactions directly with live *C. albicans* hyphae. This expands the utility of the assay beyond the relatively artificial clusters of yeast or microbe-like particles (zymosan).^10,13^ Furthermore, direct study of the swarming response to hyphae allows us to understand what mechanisms and observations translate across both yeast clusters and hyphae and which may be unique to specific targets. As examples, we found that LTB_4_ receptor antagonists caused significant, but not complete, reductions in swarm size against hyphae, which is consistent with previous results in human neutrophils against yeast or zymosan clusters as well. This phenotype is unique to human cells, as disruptions to LTB_4_ signaling cause complete disruptions to swarming in mouse neutrophils.^5,33^ In contrast, we found major differences in NET profiles when swarming was compared between yeast clusters and hyphae, with the yeast clusters resulting in more SYTOX Green signal within their swarms. Previously, we had suggested that NET release within the swarms was a way for the neutrophils to try to restrict any microbes that survived past the initial release of antimicrobials.^13^ We suggest that this may be the reason for the difference in NETs here, as the yeast clusters had significantly more surviving fungi to need restriction when compared with the hyphae, which were more effectively killed by the swarms. In keeping with this, disruption of NETs resulted in large disruptions to fungal restriction against the yeast clusters, while they made only small disruptions to the restriction of hyphae.^13^

Early swarming events have been a major focus of study, specifically the events directly following when pioneer neutrophils encounter target pathogens or area of injury. Here, we provide unprecedented detail into the interactions between the pioneer neutrophil and individual fungal hyphae by leveraging the fluorescent probe Calbryte as a reporter for calcium influx, cell activation, and signaling.^28^ Calcium signaling patterns have been examined in neutrophils responding to sterile wounding in an in vivo zebrafish model.^9^ This calcium flux was preferentially tied to LTB_4_ synthesis and the calcium signaling waves seen in mouse or human neutrophils in response to yeast clusters were also LTB_4_ dependent, consistent with LTB_4_'s role as a critical mediator of swarming recruitment.^9,11,28^ Due to the differences between sterile wounding, yeast clusters, and fungal hyphae as targets, it will be important to investigate how these in vitro calcium signaling results translate to in vivo models of infection.^9,11,28^

The trigger for swarming has been proposed to involve a size threshold and previous work showed that zymosan or yeast clusters required a minimum area to reliably induce swarming responses.^10,13^ Our data build on this idea of a size threshold and, by utilizing hyphae, which are a single large entity as opposed to a cluster, we directly quantified neutrophil stretching before triggering swarming. While it is difficult to directly compare with the size threshold established previously (i.e. target cluster size), as this is neutrophil length, our data provide further evidence and detailed insights for swarming as a response against large pathogens, opposite the axis of phagocytosis. Furthermore, we show that a single neutrophil is sufficient to rapidly initiate a swarming response after encountering a single hyphal segment, something that would have been difficult to demonstrate in previous versions of this assay.^10,13^ There is heterogeneity in this response though, so future work will be required to elucidate the specific factors that influence the speed and amount of stretching required before neutrophils make the decision to initiate swarming. Together, these highlight some unique insights that were enabled by directly using hyphae as initiating targets and build our understanding of the early events in the swarming response.

MPO played an important role in the ability of the swarm to restrict fungal growth, which is in line with its overall importance to antifungal defense and previous results with swarming against yeast clusters.^13,34^ While MPO can contribute to NET production, our results here show minimal difference in NETs during inhibition of MPO by 4-ABAH, suggesting that its major antifungal contribution during swarming against hyphae is probably direct production of antimicrobials. Interestingly, we show that MPO inhibition also results in significantly larger swarm sizes. Previous work has shown that ROS plays an important role in regulating the dynamics of the swarm, with ROS inhibition by genetic deficiency or chemical inhibition of the NADPH oxidase causing larger signaling waves and larger swarm sizes.^13,28^ These results demonstrate that MPO products, not just direct NADPH oxidase products, can also play a role in the regulation of swarming. Recent work provided visual insights into the localization of both OH and HClO within the swarm, and our results here suggest that both are relevant for the regulatory role of ROS in swarming dynamics.^14^

We found that SYK signaling play critical roles in the initiation of effective swarming against *C. albicans* hyphae, as blocking completely prevents swarming and results in uncontrolled fungal growth. SYK signaling appears to be a master regulator for neutrophil responses to fungi, as swarming to yeast clusters as well as traditional functions like phagocytosis and ROS production are also compromised during SYK inhibition.^30,35^ It is therefore likely that SYK signaling is required for the earliest phases of neutrophil/pathogen interactions during swarming, such as initial recognition of the pathogen via cell surface receptors.^36^ In support of this, our Calbryte staining experiments show that SYK signaling often completely blocks neutrophils from attaching and triggering signaling waves or swarming, and even when signaling is triggered, the distance travelled is significantly reduced. While it is possible that the interpretation of this data could be complicated by off-target effects from the chemical inhibitor, our phenotype is in line for that seen in genetic knockout Hoxb8-derived neutrophils deficient in SYK, which suggests that the critical importance of SYK to swarming is a real phenotype.^30^

We also found that BTK signaling plays an important role in swarming responses to *C. albicans* hyphae, as it resulted in a significant reduction of swarm formation and fungal growth restriction. This is in line with recent results showing that BTK inhibition also reduces the swarming response against *A. fumigatus* conidia, as well as results showing the overall importance of BTK signaling to neutrophil functions and infection outcomes in vivo.^32,37^ These results extend the importance of BTK signaling to swarming against *C. albicans*. Interestingly, the defects caused by BTK signaling were not as pronounced as that seen during SYK inhibition, as BTK inhibited neutrophils trended toward forming larger clusters against the hyphae and controlled fungal growth significantly better than SYK inhibited cells. Our results from Calbryte experiments show that both SYK and BTK inhibition also reduce the distance that signaling waves are transmitted from the pioneer neutrophils. This results in neutrophils being recruited from a significantly reduced distance from the fungi, and unsurprisingly, the formation of smaller, ineffective swarms. SYK inhibition in particular seems to disrupt very early events in swarm initiation, as a number of hyphae did not trigger swarms despite being surround by neutrophils, which was not seen during BTK inhibition. This additional defect may be partially why SYK inhibition results in more severe disruptions than BTK inhibition. Further experiments will be required to elucidate the reasons for this difference, but it may provide insights into the hierarchy of these mediators and their role in regulating swarming responses.

Finally, we found that blocking CD18 resulted in smaller swarms and increased fungal growth. Interestingly, this defect in swarm growth did not appear tied to initiation events, as we found that early events like neutrophil stretching, time from attachment to signaling, and wave size were not significantly different from controls. Instead, it appeared that swarms were not able to grow significantly past the “first layer” of neutrophils that arrived and covered hyphae. This defect appeared to be specific to swarming, as traditional phagocytosis, ROS production, and killing assays against *C. albicans* yeast did not show the same significant defects. This phenotype closely resembles results seen previously in vivo, in which mouse cells deficient in this receptor were unable to migrate into the growing swarm cluster, instead staying at the periphery.^5^

This phenotype was not found to be tied to known traditional CD18 partners as blocking CD11a, CD11b, or CD11c did not result in this phenotype. Interestingly, previous work has demonstrated that CD11a would be expected to exhibit defects as well; however, they were not seen in this system.^5^ The results of CD11b blocking depended on the clone of antibody used, with results ranging from no significant or minor changes to major phenotype changes in the case of VIM12. Interestingly, this was only true during swarming, as no significant changes were seen to phagocytosis or ROS production against yeast by flow cytometry. The reasons for these differences are unclear and genetic deficiency may be needed for clearer insights into the role of CD11b during swarming. It is also not clear why our results differ from those seen in vivo for CD11a and CD11b, though it could be due to differences in in vitro versus in vivo environments, differences in the requirements during swarming with mouse and human cells, or differences in the requirements for these receptors depending on the target (pathogen vs. sterile wounding).^5,10^ Further experiments will be needed to further explore and elucidate these mechanisms. Despite these differences, our results show that the phenotype seen during CD18 blocking is similar across these in vitro and in vivo model systems and across mouse and human cells and that it is unique to CD18, as blocking with CD11a, CD11b, or CD11c did not show similar results. Furthermore, our results show that the ability of the swarm to continue to grow past a single layer of cells is critical for it to reach maximum antimicrobial potential, highlighting the importance of cell-cell communication in swarm function. CD18 function has previously been found to be important for the killing of *A. fumigatus* hyphae, though swarming specifically was not assessed, suggesting that this mechanism may be relevant across fungal species.^38^

Together, we present an optimized microscale array to study human neutrophil swarming directly against pregrown *C. albicans* hyphae. We provided detailed insights into the early events of initiation by pioneer neutrophils after they encounter hyphae as well as into the molecular mechanisms that regulate this process and enable it to effectively restrict fungal growth. Importantly, we highlight mechanisms and observations that translate between the arrays of biologically relevant hyphae and those made with previous versions of the assay, like the role of LTB_4_ in human neutrophil swarming and the critical role of SYK signaling, while also identifying some which are unique, such as differences in NETosis.^10,13,30^ By carefully identifying and leveraging the advantages of each target and model system, we will be able to rapidly characterize the molecular mechanisms that regulate swarming and elucidate its role during infection and inflammation in the future. These swarming arrays are also well positioned to directly test the swarming capabilities of the neutrophils of at-risk patient populations, but the potential of this system is held back by our lack of understanding of the molecular mechanisms that drive the process.^13,39–42^ By elucidating the detailed mechanisms of swarming, we expect to identify the key receptors and pathways which control this process. This will allow us to specifically screen patient cells for important defects that may render them susceptible to infection and to design novel therapeutic strategies to harness the power of neutrophils and swarming to treat or prevent patient infections in the future.

## Supplementary Material

qiaf082_Supplementary_Data

## Data Availability

Data will be made available upon reasonable request. Submit requests to Dr. Hopke.
