# Supplementary material for: Caging giants: Characterizing the molecular mechanisms of neutrophil swarming against Candida albicans hyphae
Source: J Leukoc Biol. 2025 Jun 6;117(7):qiaf082. doi: 10.1093/jleuko/qiaf082 (PMC12256248; doi:10.1093/jleuko/qiaf082)
Supplement: qiaf082_Supplementary_Data [file qiaf082_supplementary_data.zip › Supplemental Information v3.pdf]

Supplementary Information:

Caging Giants: Characterizing the molecular mechanisms of neutrophil swarming against *Candida albican*s hyphae

Tasha K. Phillips[1], Kelsey Lawson[2], Tammy R. Ozment[3,4], Allison Scherer[2], Alex Hopke[1,4*]
Affiliations:
1: *East Tennessee State University Quillen College of Medicine, Department of Biomedical Sciences, Johnson City, United States of America*
2: University of Virginia at Wise, Department of Natural Sciences, Wise, United States of America
3: East Tennessee State University Quillen College of Medicine, Department of Surgery, Johnson City, United States of America
4: Center of Excellence in Inflammation, Infectious Disease and Immunity, East Tennessee State University, Johnson City, United States of America

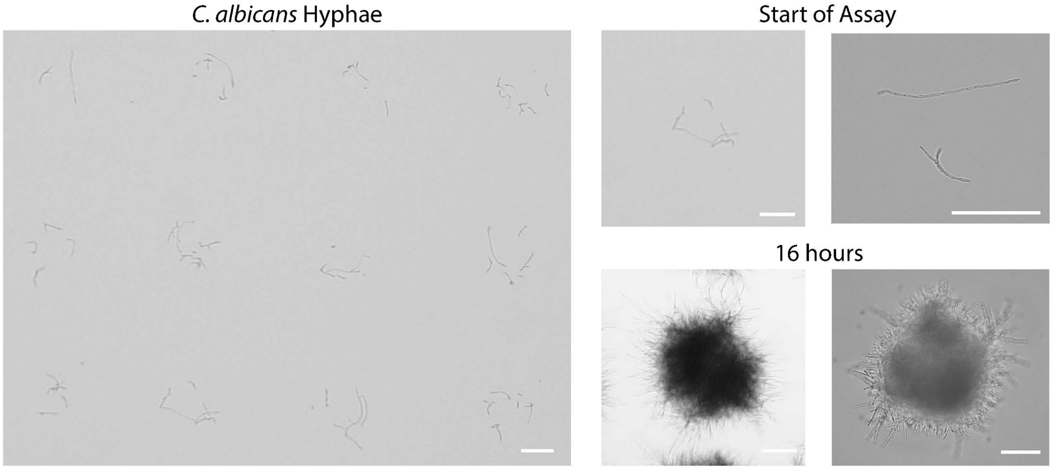

Supplementary Figure 1: Patterned Arrays of Live *C. albicans* Hyphae

Live *C. albicans* hyphae were patterned on printed arrays of poly-l-lysine. Individual spots had small numbers of individual hyphae. Live hyphae were able to grow and invade the surrounding space radiating out from their original location, shown at 16 hours after the start of the assay. Scale bar represents 100 µm.

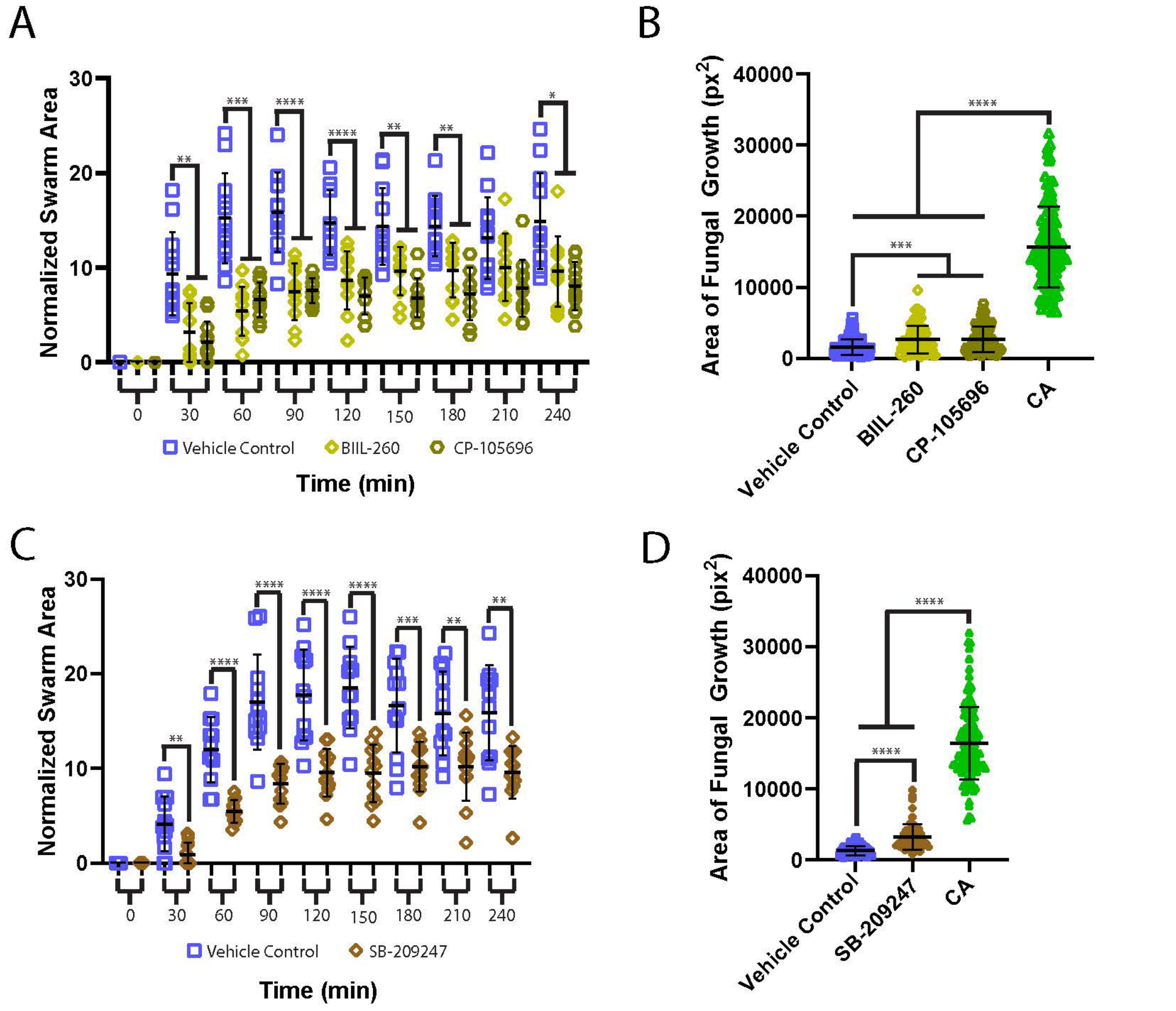

Supplementary Figure 2: Disrupting LTB_4 signaling significantly impacts swarming and fungal restriction

Human neutrophils were treated with the LTB4 receptor antagonists BIIL-260, CP-105696 or SB-209247 or vehicle control and added to the arrays of fungal hyphae. (**A**) The area of the swarms at the indicated time was quantified and normalized to the amount of starting hyphae for vehicle control or BIIL-260 or CP-105696 treated neutrophils. N=12 swarms across 3 donors. (**B**) The remaining fungal growth was quantified after 16 hours of incubation with human neutrophils treated with vehicle or the same drugs as in A or media only. N≥ 118 spots across 3 donors. (**C**) The area of the swarms at the indicated time was quantified and normalized to the amount of starting hyphae for vehicle control SB-209247 treated neutrophils. N= 12 swarms across 3 donors. (**D**) The remaining fungal growth was quantified after 16 hours of incubation with human neutrophils treated with vehicle control, SB-209247 or media only. N≥ 124 spots across 3 donors. * p≤ 0.05, ** p≤0.01, *** p≤0.001 **** p<0.0001 by Students T-test, Mann-Whitney test or Kruskal-Wallis test. Error bars represent standard deviation.

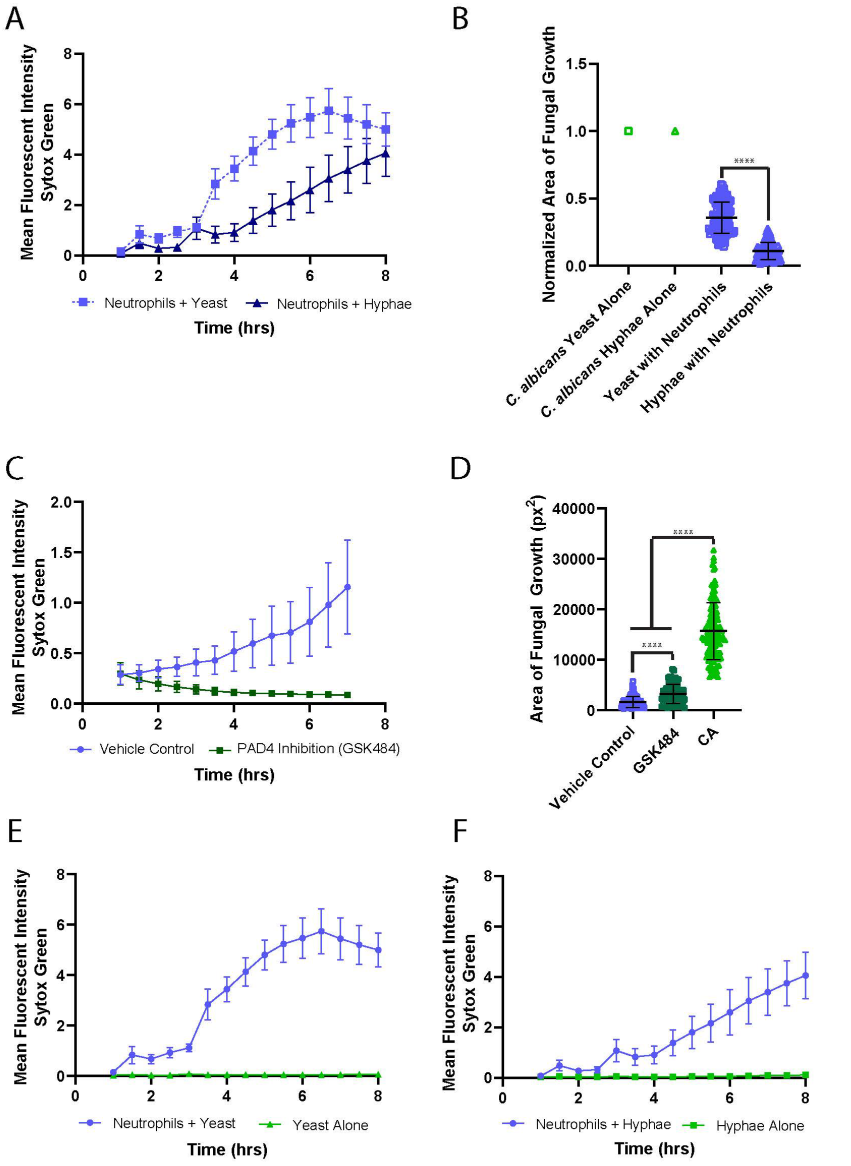

Supplementary Figure 3: Yeast clusters and hyphae elicit unique NET profiles

Human neutrophils were added to the arrays of live yeast clusters or fungal hyphae along with Sytox green. (**A**) The mean fluorescent intensity of the sytox green was quantified with each swarm. N=12 swarms across 3 donors. (**B**) The remaining fungal growth was quantified after 16 hours of incubation with human neutrophils for yeast clusters or hyphae. Results were normalized to their respective media only controls. N≥ 107 spots across 3 donors. (**C**) The mean fluorescent intensity of the sytox green was quantified with each swarm after treatment with vehicle control of PAD4 inhibitor GSK484. N= 12 swarms across 3 donors. (**D**) The remaining fungal growth was quantified after 16 hours of incubation with human neutrophils treated with vehicle control, GSK484 or media only. N≥ 118 spots across 3 donors. (**E**) The mean fluorescent intensity of the sytox green was quantified with each yeast cluster with or without the addition of neutrophils. N= 12 swarms. (F) The mean fluorescent intensity of the sytox green was quantified with each hyphae, with or without the addition of neutrophils. N=12 swarms. **** $p<0.0001$ by Kruskal-Wallis test. Error bars represent standard error of the mean for A, C and E and standard deviation for B, D and F.

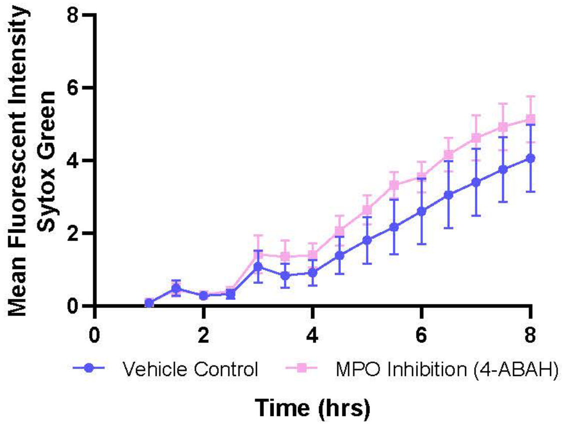

Supplementary Figure 4: Inhibition of MPO does not significantly change NET release during swarming against hyphae

Human neutrophils were treated with vehicle control or 4-ABAH and added to the arrays of fungal hyphae. The mean fluorescent intensity of sytox green was quantified at each swarm over time. Error bars represent standard error of the mean.

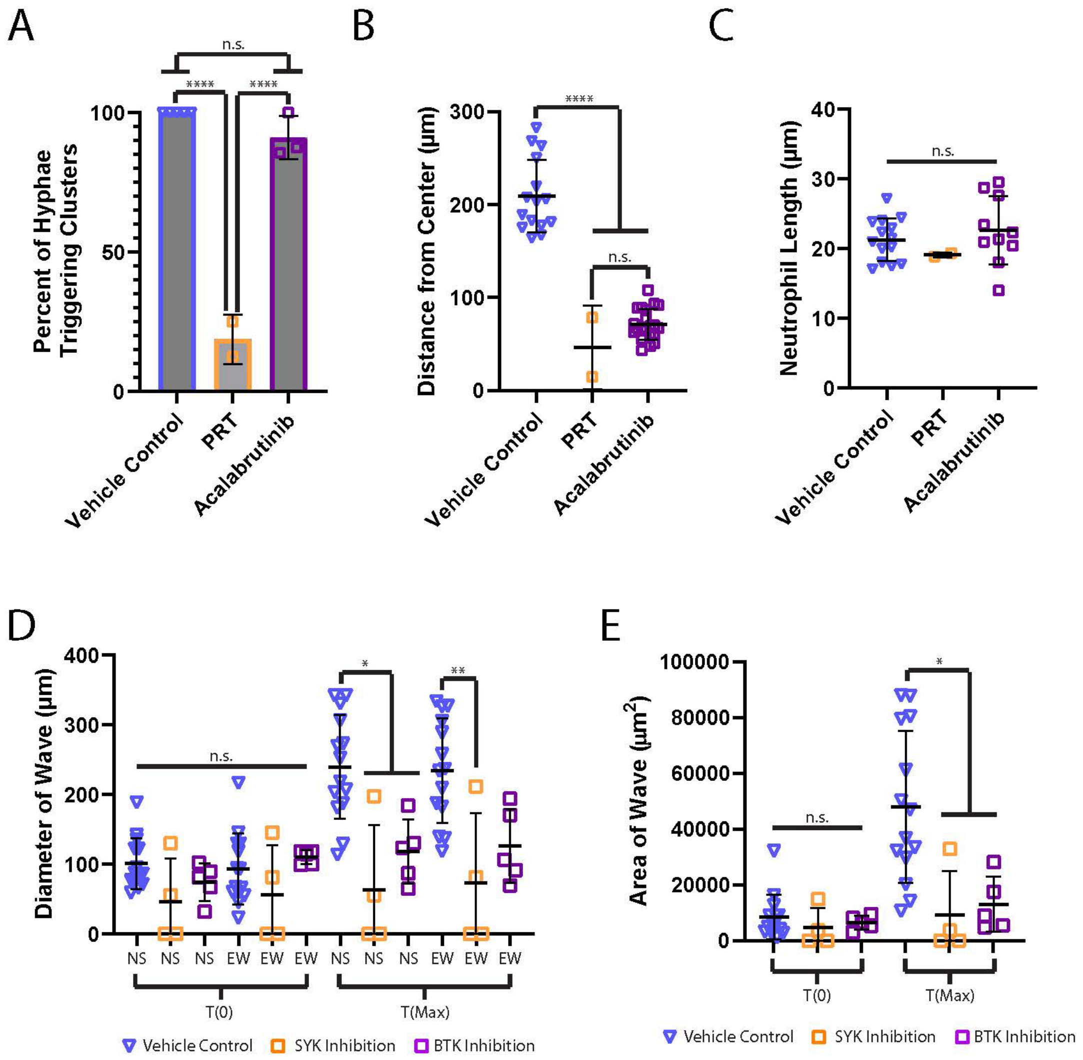

Supplementary Figure 5: Early interactions with *C. albicans* are disrupted by inhibition of SYK or BTK.

Purified human neutrophils were stained with Calbryte, treated with vehicle control (DMSO), PRT or Acalabrutinib and then added to *C. albicans* hyphal arrays. (**A**) The percent of hyphae spots that triggered neutrophil clustering was quantified. N≥14 spots across 3 donors, except for PRT which is from 2 donors. (**B**) The maximum distance from hyphae from which neutrophils migrated into the swarm was quantified. N≥15 swarms across 3 donors for vehicle and Acalabrutinib, N=2 swarms across 2 donors for PRT. (**C**) The length the neutrophil stretched before the Calbryte signal spreads to neighboring cells was quantified. N≥10 neutrophils across 3 donors for vehicle and Acalabrutinib, N=2 neutrophils across 2 donors for PRT. (C) The diameter of the Calbryte positive signaling wave was measured at the first frame of wave spread and at the frame where the wave spread to its maximum size. The diameter was measured for both the North/South axis (NS) and the East/West axis (EW). N≥4 waves across at least 2 donors. (**E**) The area covered by each wave in the first visible frame (time start) and at its maximum size (time final) are shown. N≥ 4 waves across at least 2 donors. n.s. is not significant, * p≤ 0.05, ** p≤0.001, **** p<0.0001, by one-way ANOVA or Kruskal-Wallis tests. Error bars represent standard deviation.

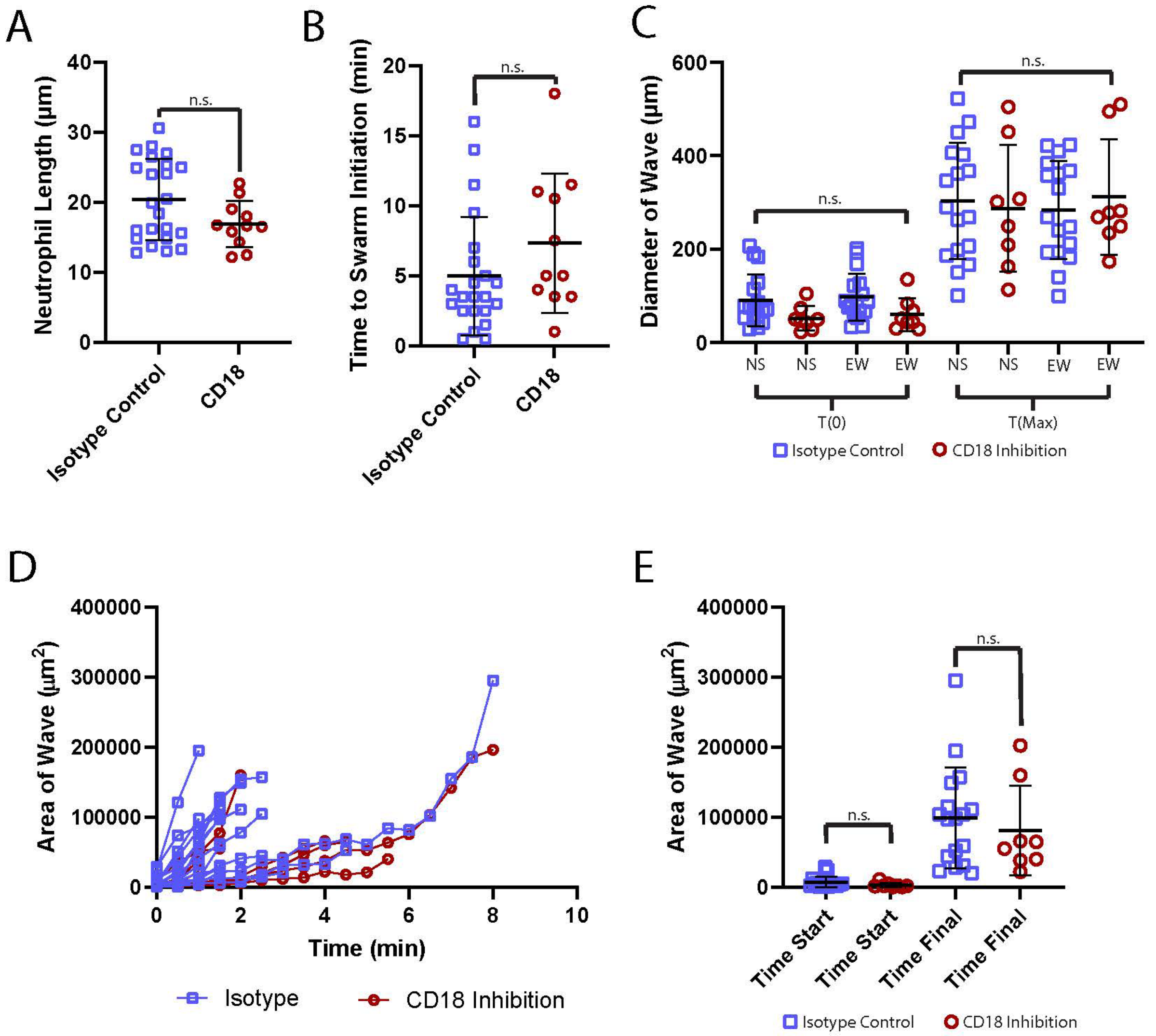

Supplementary Figure 6: Early interactions with *C. albicans* are not changed by CD18 blocking.

Purified human neutrophils were stained with Calbryte and then added to *C. albicans* hyphal arrays. (A) The length the neutrophil stretched before the Calbryte signal spreads to neighboring cells was quantified. N= 23 neutrophils across 3 donors for isotype and N=11 neutrophils across 2 donors for CD18 blocking. (**B**) The time from the attachment of the neutrophil to the hyphae until the Calbryte signal spread to neighboring cells was quantified. N= 22 neutrophils across 3 donors for isotype and N=11 neutrophils across 2 donors for CD18 blocking. (C) The diameter of the Calbryte positive signaling wave was measured at the first frame of wave spread and at the frame where the wave spread to its maximum size. The diameter was measured for both the North/South axis (NS) and the East/West axis (EW). N≥8 waves across at least 2 donors. (**D**) The area covered by the wave was calculated from the first frame it spread from the pioneer neutrophil until its maximum size. Each line represents an individual wave. (**E**) The area covered by each wave in the first visible frame (time start) and at its maximum size (time final) are shown. N≥ 8 waves across at least 2 donors. n.s. is not significant by Mann-Whitney or Kruskal-Wallis tests. Error bars represent standard deviation.

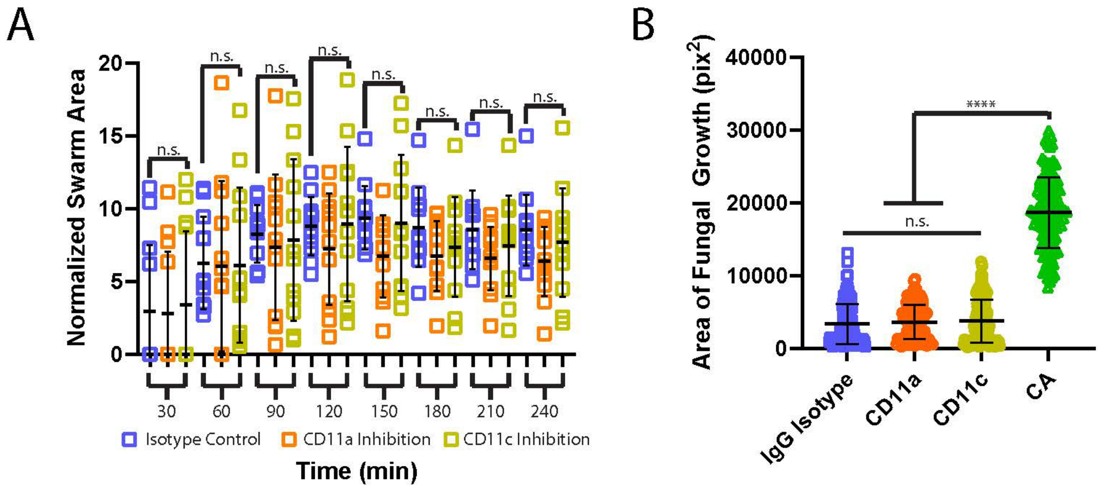

Supplementary Figure 7: Blocking of CD18 binding partners Cd11a or CD11c do not significantly impact swarming and fungal restriction

Human neutrophils were treated with blocking antibody for CD11a, CD11c or isotype antibody and added to the arrays of fungal hyphae. (**A**) The area of the swarms at the indicated time was quantified and normalized to the amount of starting hyphae for isotype control or CD11a or CD11c inhibited neutrophils. N≥ 12 swarms across 3 donors. (**B**) The remaining fungal growth was quantified after 16 hours of incubation with human neutrophils treated with isotype antibody, CD11a or CD11c blocking antibody or media only. N≥ 111 spots across 3 donors. n.s. is not significant, **** $p<0.0001$ by Kruskal-Wallis test. Error bars represent standard deviation.

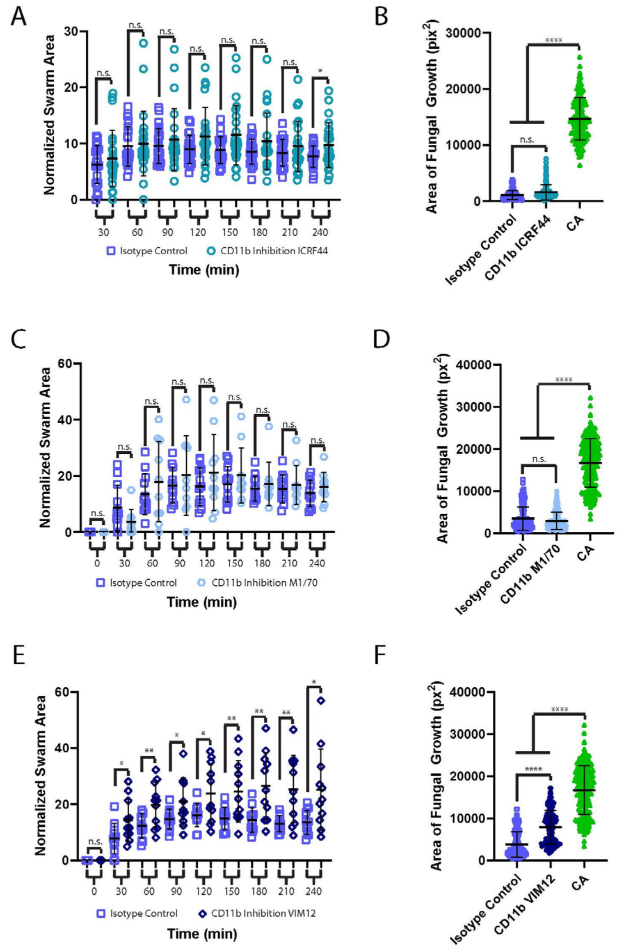

Supplementary Figure 8: The impact of blocking of CD11b on swarming and fungal restriction depends on the antibody clone

Human neutrophils were treated with blocking antibody for CD11b of three different clones (ICRF44, M1/70 or VIM12) or isotype antibody and added to the arrays of fungal hyphae. (**A**) The area of the swarms at the indicated time was quantified and normalized to the amount of starting hyphae for isotype control or CD11b clone ICRF44 inhibited neutrophils. N≥ 22 swarms across 6 donors. (**B**) The remaining fungal growth was quantified after 16 hours of incubation with human neutrophils treated with isotype antibody, CD11b clone ICRF44 blocking antibody or media only. N≥ 121 spots across 3 donors. (**C**) The area of the swarms at the indicated time was quantified and normalized to the amount of starting hyphae for isotype control or CD11b clone M1/70 inhibited neutrophils. N≥ 10 swarms across 3 donors. (**D**) The remaining fungal growth was quantified after 16 hours of incubation with human neutrophils treated with isotype antibody, CD11b clone M1/70 blocking antibody or media only. N≥ 197 spots across 4 donors. (**E**) The area of the swarms at the indicated time was quantified and normalized to the amount of starting hyphae for isotype control or CD11b clone VIM12 inhibited neutrophils. N≥ 11 swarms across 3 donors. (**F**) The remaining fungal growth was quantified after 16 hours of incubation with human neutrophils treated with isotype antibody, CD11b clone VIM12 blocking antibody or media only. N≥ 197 spots across 4 donors. n.s is not significant, * p≤ 0.05, ** p≤0.001, **** p<0.0001 by Students T-test, Mann-Whitney or Kruskal-Wallis test. Error bars represent standard deviation.

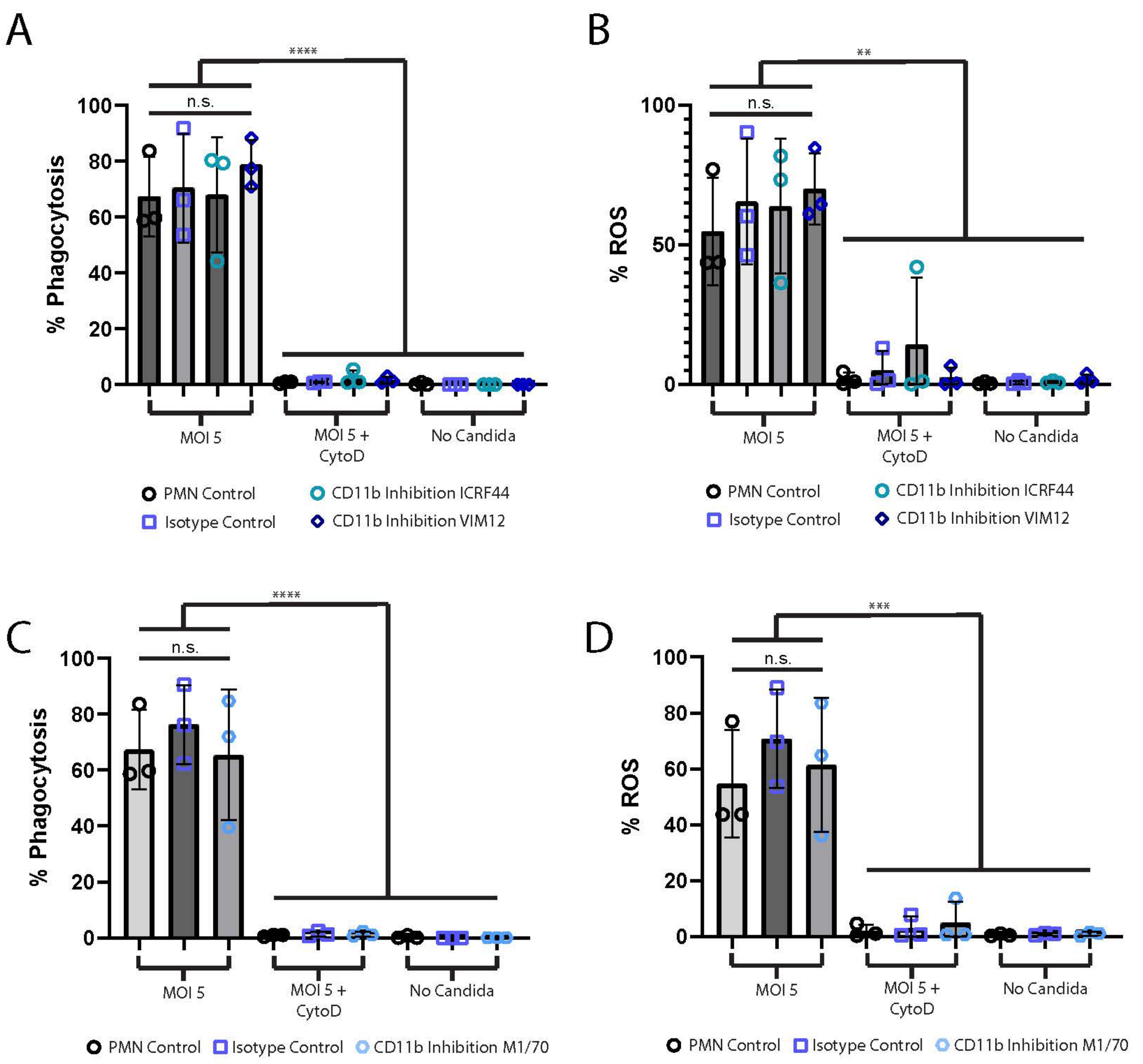

Supplementary Figure 9: CD11b inhibition does not significantly impact phagocytosis or ROS production against *C. albicans* yeast in this assay

Human neutrophils were left untreated (PMN control) or treated with one of CD11b blocking antibodies (ICRF44, VIM12, M1/70) or isotype control. (**A**) Neutrophils were co-incubated with *C. albicans* yeast (SC5314-iRFP strain) at MOI 5 for 20 min, then labeled with CD66b-PE. Phagocytosis events were measured via flow cytometry from the CD66b-PE positive cell population that also had iRFP fluorescence. Data is shown for the antibodies with an IgG1 isotype control. (**B**) ROS events were measured from CD66b-PE positive cells that were also FITC (DHR-123) positive. Data is shown for antibodies with an IgG1 isotype control. (**C**) Phagoctosis events were measured as outlined above. Data is shown for antibodies with an IgG2b control. (**D**) ROS events were measured as outlined above. Data is shown for antibodies with an IgG2b isotype control. Cytochalasin D was used as a negative control for phagocytosis events. Data were pooled from 3 independent donors for all experiments. n.s is not significant, **p<0.01, ***p<0.001, **** p<0.0001 by Kruskal-Wallis test. Error bars represent standard deviation.

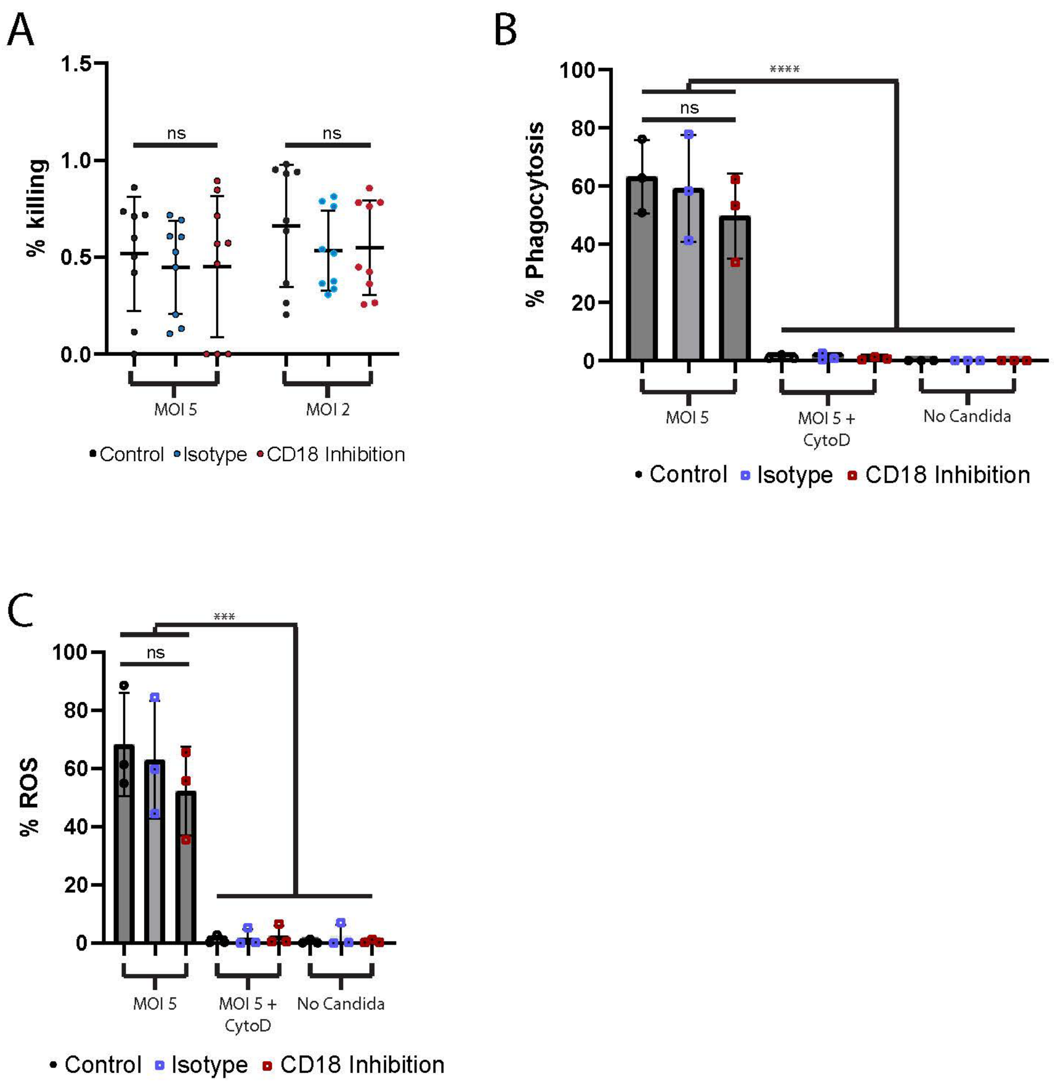

Supplementary Figure 10: CD18 inhibition does not significantly impact phagocytosis, ROS production or killing of *C. albicans* yeast in this assay

Human neutrophils were treated with blocking antibody for CD18 or isotype control. (**A**) Neutrophils were co-incubated with a multiplicity of infection (MOI) of 5 or 2 with *C. albicans* yeast for 2 hrs. Neutrophils were then lysed, and *C. albicans* metabolic activity was measured using PrestoBlue. Data represents results from 3 independent experiments. (**B**) Neutrophils were co-incubated with *C. albicans* yeast (SC5314-iRFP strain) at MOI 5 for 20 min, then labeled with CD11b-PE. Phagocytosis events were measured via flow cytometry from the CD11b-PE positive cell population that also had iRFP fluorescence. ROS events were measured from CD11b-PE positive cells that were also FITC (DHR-123) positive. Cytochalasin D was used as a control for phagocytosis events. Data were pooled from 3 independent experiments. n.s is not significant, ***p<0.001, **** p<0.0001 by Kruskal-Wallis test. Error bars represent standard deviation.
